# Development of a multiparameter flow cytometric assay as a potential biomarker for homologous recombination deficiency in women with high-grade serous ovarian cancer

**DOI:** 10.1186/s12967-015-0604-z

**Published:** 2015-07-22

**Authors:** Jung-Min Lee, Nicolas Gordon, Jane B Trepel, Min-Jung Lee, Minshu Yu, Elise C Kohn

**Affiliations:** 1 Women’s Malignancies Branch, Center for Cancer Research, National Cancer Institute, 10 Center Dr. MSC1906, Building 10, Room 12N/226, Bethesda, MD 20892-1906 USA; 2 Developmental Therapeutics Branch, Center for Cancer Research, National Cancer Institute, Bethesda, MD 20892 USA

**Keywords:** Ovarian cancer, PARP inhibitor, Biomarkers, Flow cytometry, Peripheral blood mononuclear cells, γH2AX, MRE11, RAD51

## Abstract

**Objectives:**

PARP inhibitors (PARPi) are a novel class of drugs with activity in patients with acquired or germline homologous recombination (HR) deficiency-associated high-grade serous ovarian cancer (HGSOC). We hypothesized that measuring γH2AX as an indicator of DNA double-strand breaks (DSB), and MRE11 or RAD51 as an indicator of DSB repair, would reflect HR status and predict response to PARPi-based therapy. Our aim was to develop and use high-throughput multiparametric flow cytometry to quantify γH2AX with MRE11 or RAD51 in PBMCs as a readily available surrogate.

**Methods:**

Healthy donor PBMCs were used for assay development and optimization. We validated induction of γH2AX, MRE11 and RAD51 by staining with fluorophore-conjugated antibodies. The multiparameter flow cytometric method was applied to PBMC samples from recurrent HGSOC patients who were treated with PARPi, olaparib and carboplatin.

**Results:**

Stimulation was necessary for quantification of a DNA damage response to olaparib/carboplatin in healthy donor PBMCs. The flow cytometric protocol could not distinguish between cytoplasmic and nuclear RAD51, erroneously indicating activation in response to injury. Thus, MRE11 was selected as the marker of DSB repair. PBMCs from 15 recurrent HGSOC patients were then examined. Patients who did not respond to PARPi therapy had a significantly higher pre-treatment level of γH2AX (p = 0.01), and a higher ratio of γH2AX/MRE11 (11.0 [3.5–13.2] v. 3.3 [2.8–9.9], p < 0.03) compared with responders.

**Conclusions:**

We successfully developed and applied a multiparameter flow cytometry assay to measure γH2AX and MRE11 in PBMCs. Prospective studies will be required to validate this surrogate biomarker assay as a potential predictive biomarker of PARPi-based therapy.

**Electronic supplementary material:**

The online version of this article (doi:10.1186/s12967-015-0604-z) contains supplementary material, which is available to authorized users.

## Background

Poly(ADP-ribose) polymerase inhibitors (PARPi) are a novel class of drugs with promising activity in patients with deleterious germline *BRCA1* and *BRCA2* mutation (gBRCAm)-associated ovarian and breast cancers, and sporadic high-grade serous ovarian cancer (HGSOC) [1, 2]. The PARPi, olaparib was recently approved by US Food and Drug Administration for heavily pretreated gBRCAm-associated ovarian cancer. Reported response rates (RRs) are ~40% in gBRCAm and 24% in *BRCA1/2* wild-type (BRCAwt) ovarian cancer patients [1]. The susceptibility of patients with gBRCAm-associated ovarian cancer to DNA damaging agents, including PARPi, has validated gBRCAm as a predictive biomarker for PARPi response [3]. However, at least half of gBRCAm biomarker-positive women do not respond well to PARPi and many BRCAwt HGSOC women do respond. The challenge remains to identify, develop, and validate biomarkers to apply within this HGSOC patient population to predict more accurately who will benefit from PARPi therapies.

One of the key components in DNA damage repair is the histone protein H2AX, which becomes rapidly phosphorylated on serine 139 to form γH2AX, a process occurring at nascent DNA double-strand breaks (DSBs) [4]. This creates a focus for accumulation of DNA repair and chromatin remodeling proteins. γH2AX has been proposed as a biomarker of DSBs in response to damage. These DSBs can be immunolabeled with an antibody to 139Ser-phosphorylated H2AX, and the extent of DSBs estimated from the number of labeled nuclear foci or by measuring overall γH2AX protein levels [4]. Accumulation of γH2AX forms an injury protein/DNA complex that recruits repair proteins, including MRE11 and RAD51 [5, 6]. MRE11 binds to the damaged DNA and subsequently recruits and activates additional proteins including BRCA1, BRCA2, and RAD51 to activate the repair process [7]. RAD51 forms quantifiable nuclear immunofluorescence-detectable foci that represent the repair protein complex assembly at sites of homologous recombination (HR) [8].

There is precedent for examination of γH2AX, RAD51 and MRE11 as potential biomarkers of HR competence. γH2AX has been used as a pharmacodynamic biomarker of DNA damaging agents, measured in surrogate tissues such as plucked eyebrow-hair follicles, peripheral blood mononuclear cells (PBMCs), and has also been examined in tumor cells [9–11]. RAD51 focus formation was used to assess HR competence in HGSOC ascites primary cultures and correlated with response to PARPi in vitro [12]. MRE11 protein expression by immunohistochemistry was shown to correlate with disease-specific survival in localized invasive bladder cancer patients receiving radiotherapy [13, 14]. However, none of these are validated as a biomarker to predict clinical drug benefit, and it is possible that neither measures of damage nor measures of repair are sufficient in isolation.

PBMCs from cancer patients have been investigated as readily available surrogate sources in which to examine pharmacodynamic responses [15–17]. PBMCs from breast and lung cancer patients yielded greater in vitro accumulation of DNA damage after radiation measured by micronucleus-centromere and comet assays compared to healthy donor PBMCs, possibly reflecting tumor genomic instability and indicating PBMCs can serve as a surrogate tumor [17–19]. Our aim was to quantify DNA damage and repair in PBMCs from HGSOC patients using a rapid, high-throughput quantitative measure, such as flow cytometry, that could be applicable broadly. We hypothesized that a gauge incorporating both DNA damage and repair may more accurately characterize susceptibility to PARPi-based therapy. Here, we demonstrate the development and application of a multiparameter flow cytometric method measuring γH2AX and MRE11 in PBMCs from women with HGSOC who received PARPi therapy.

## Methods

### Healthy donor PBMC collection, isolation, stimulation, and treatment

PBMCs from healthy donors were obtained from the NIH Clinical Center Department of Transfusion Medicine under their Institutional Review Board (IRB)-approved and consented healthy donor program. PBMCs were spun in cell separation tubes for 30 min at 1,500×*g* within 2 h of venipuncture, and isolated cells were incubated overnight in serum-containing medium. Viability was determined by trypan blue exclusion prior to use. Where indicated, phorbol myristate acetate (PMA, 50 ng/mL) and ionomycin (1 μg/mL) or DMSO vehicle control (<0.05%) were added to stimulate donor PBMCs for 4 h before treatment with carboplatin 50 μM and olaparib 10 μM (Selleck Chemicals, Houston, TX, USA) or DMSO (<0.05%). Where indicated donor PBMCs were frozen in medium containing 90% FBS/10% DMSO and placed at −80°C overnight, with a parallel set designated for flow cytometry without first freezing. Frozen cells were thawed at room temperature, washed once in PBS, and seeded onto 24-well plates in serum-containing RPMI-1640 medium. Paired freeze/thaw and direct stained PBMC sets were from the same donor.

### Flow cytometry

A standard validated intracellular staining protocol was used [20]. PBMCs were fixed overnight in 0.4% paraformaldehyde at 4°C and subsequently stained. Primary antibodies for single-color flow cytometry included rabbit anti human-γH2AX preconjugated with Alexa Fluor 488 (1:200; Abcam, Cambridge, MA, USA), rabbit anti-human MRE11 (1:100; Cell Signaling Technology, Beverly, MA, USA), and rabbit anti-human RAD51A (1:100; Abcam). Alexa Fluor 488 conjugated goat anti-rabbit IgG (1:500; Invitrogen, Grand Island, NY, USA) was used as a secondary antibody for MRE11 and RAD51. The single-color flow protocol was used during experiments to optimize the stimulation conditions, after which a dual-stain protocol was optimized using antibodies from different species: rabbit anti-human RAD51A (1:100; Abcam) and mouse anti-human γH2AX (1:200; Abcam), and rabbit anti-human MRE11 (1:100; Cell Signaling Technology). Alexa Fluor 488 goat anti-rabbit IgG was used as the secondary antibody for MRE11 and RAD51. Alexa Fluor 647 goat anti-mouse IgG (1:500; Abcam) was the secondary antibody for γH2AX. Negative controls of dual-stain flow cytometry used anti-rabbit IgG isotype (Cell Signaling) and anti-mouse IgG isotype controls (Abcam) at the same dilutions as the associated primary antibodies. Unstained cells were subjected to the same treatments and detection methods as stained cells without the use of primary or secondary antibodies. PBMCs were examined using a MACSQuant Analyzer (Miltenyi Biotec; Bergisch Gladbach, DE, USA) and the median fluorescence intensity (MFI) determined by FlowJo v.X.0.6 (Treestar; Ashland, OR, USA) as reported [20]. All data for the development assays are the result of three independent experiments and are presented as mean ± SEM. Patient samples were analyzed once with a minimum of 10,000 viable cells examined per data point. Dead cells were excluded using the LIVE/DEAD Fixable Aqua Dead Cell Stain Kit (Life Technologies; Carlsbad, CA, USA).

### Immunofluorescence confocal microscopy (IF)

Healthy donor PBMCs designated for IF were treated in parallel with those designated for flow cytometry throughout stimulation and drug exposure. Cells were cytospun onto silylated slides (CEL Associates; Pearland, TX, USA) and were stained with mouse anti-human γH2AX (1:200, Abcam) and rabbit anti-MRE11 (1:200, Abcam), and rabbit anti-RAD51 (1:50, Santa Cruz Biotechnology; Santa Cruz, CA, USA) using our standard laboratory immunofluorescence protocol [21]. Alexa Fluor 488 goat-anti mouse IgG and Alexa Fluor 488 goat-anti rabbit IgG (1:500, Invitrogen) were used for IF detection. The slides were mounted with Vectashield mounting medium with DAPI (Vector Labs; Burlingame, CA, USA) and images were collected as Z stacks using a Zeiss LSM 780 confocal microscope at 1000× magnification. Images were analyzed using Zeiss LSM Image Browser version 4.2.0.121 software.

### Patients and samples

We applied the multiparameter flow cytometric method to PBMC samples from patients with recurrent HGSOC who were enrolled on one of two phase I studies conducted at the Center for Cancer Research, NCI; treatment and use of the PBMCs for this purpose was approved by the NCI IRB, and all patients had provided informed consent for such use. Patients received olaparib capsules 400 mg every 12 h (days 1–7) and carboplatin AUC4 or 5 every 21 days (NCT01445418) [22], or olaparib tablets 200 mg every 12 h (days 1–7) with carboplatin AUC4 every 21 days (NCT01237067). No more than eight combination cycles were given, and all non-progressing patients continued on twice-daily olaparib maintenance therapy until disease progression. Tumor response was assessed by imaging and physical exam every two cycles using Response Evaluation Criteria in Solid Tumors criteria. PBMCs were collected prior to treatment initiation and stored viably frozen at −80°C until use.

### Statistics

Data for the development assays were log transformed and subjected to the two-way analysis of variance. The Shapiro–Wilk test was used to test for normality after each ANOVA. The ability of the pre-treatment individual markers, measured by flow cytometry, to predict clinical response was evaluated by the Mann–Whitney test. All statistical analyses were 2-sided and performed with GraphPad Prism version 6.0 (GraphPad Software; La Jolla, CA, USA).

## Results

### PBMC stimulation is necessary for DNA damage accumulation in healthy donor PBMCs

**Figure 1:**
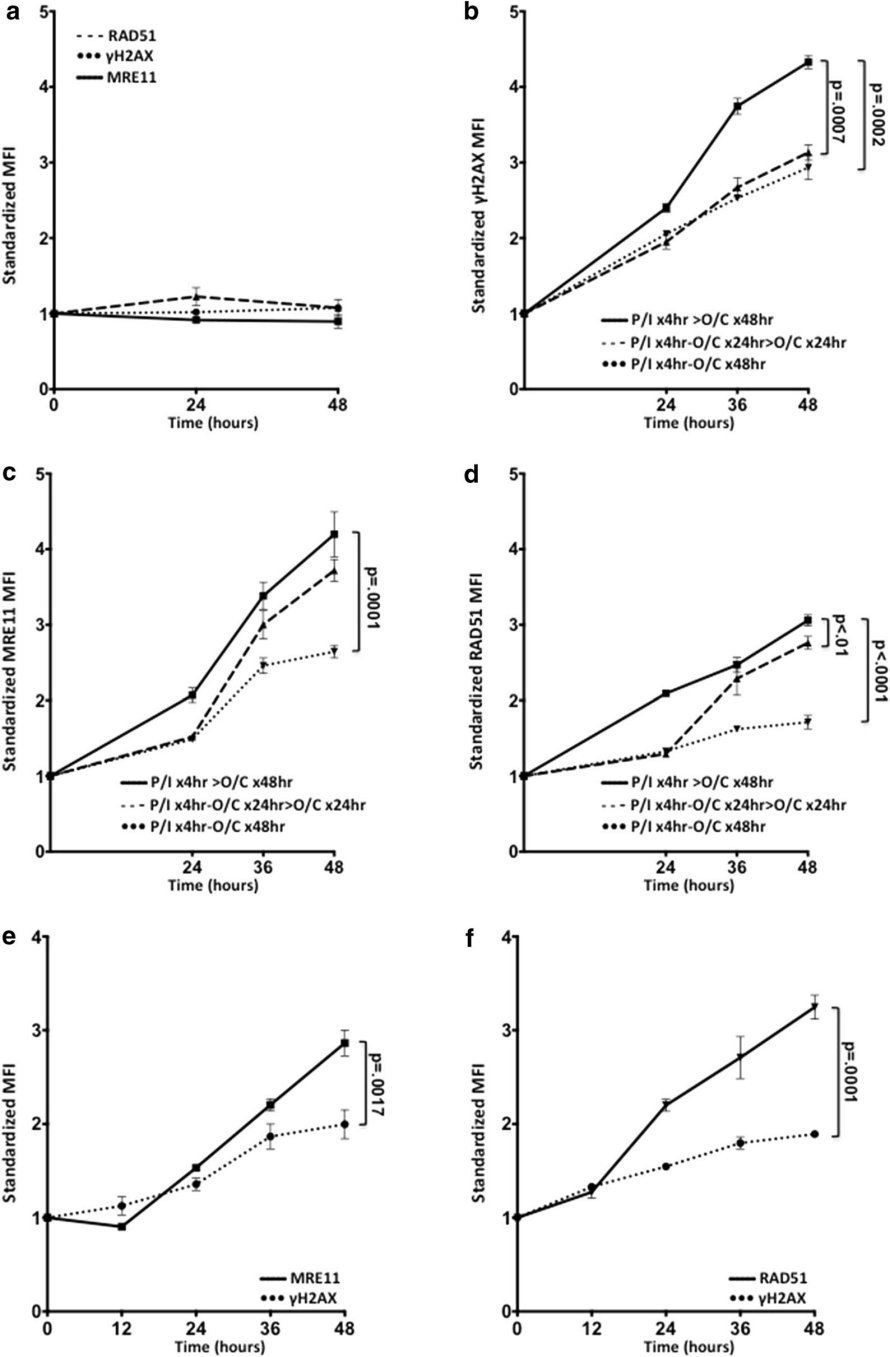
Optimization of PBMC flow cytometry method. **a** Unstimulated PBMCs. Cells were seeded and incubated overnight without PMA/I stimulation before treatment with olaparib/carboplatin (O/C) for 48 h. **b**–**d** Optimization of stimulation conditions. Condition 1: PMA/I × 4 h followed by PBS washout and subsequent O/C × 48 h (P/I × 4 h > O/C × 48 h); condition 2: PMA/I × 4 h then O/C × 24 h followed by washout and subsequent O/C × 24 h (P/I × 4 h–O/C × 24 h > O/C × 24 h); condition 3: PMA/I × 4 h followed by O/C × 48 h with no washout (P/I × 4 h–O/C × 48 h). Cells were collected after 24, 36 and 48 h of treatment before undergoing the single-stain flow cytometry protocol. **b** γH2AX; **c** MRE11; **d** RAD51. **e**, **f** Demonstration of optimized dual-stain. Cells were plated and treated according to condition 1 before undergoing the dual-stain process for MRE11 and γH2AX (**e**), or for RAD51 and γH2AX (**f**).

The use of healthy volunteer PBMCs as a surrogate resource for biomarker development and application required demonstration that they could acquire DNA damage. We thus began by modeling the treatment effect of olaparib/carboplatin (O/C) on the expression of γH2AX, MRE11, and RAD51 in healthy donor PBMCs. Figure 1a shows no induction of γH2AX, MRE11, or RAD51 expression after exposure to O/C at physiologically attainable concentrations for 48 h. Expression of the three proteins was induced in response to O/C only when PBMCs were stimulated with PMA/I (Figure 1b–d). Three conditions of stimulation were examined. In the first, PBMCs were stimulated with PMA/I, washed and then exposed to O/C for 48 h; the second introduced O/C at 4 h for 24 h, then cells were washed and re-exposed to O/C for an additional 24 h; the third introduced O/C for 48 h after the initial 4 h stimulation with no washouts. All three conditions induced statistically significant changes in γH2AX, MRE11 and RAD51 expression after 24 h (all p < 0.001). Notably, stimulation for 4 h followed by washout and subsequent O/C treatment for up to 48 h (condition 1) yielded a greater expression of γH2AX, MRE11, and RAD51 after 48 h treatment compared to the other two conditions. Flow cytometry secondary antibody and fluorophore negative controls showed no significant signal (data not shown).

### Freezing and thawing affects the number of viable cells, but not protein expression

In clinical trial settings, PBMCs are collected from patients over time after which they are, optimally, examined in a batched setting. Hence, we examined the preanalytical effect of the freeze/thaw cycle on the cell viability and flow outcome using paired PBMC samples. Paired samples from the same patient were subjected to a freeze/thaw cycle or held on wet ice, then both sets were subjected to immediate fixation and staining. Single label staining was used and dead cells were excluded and used to determine % viability as per “Methods”. There was no statistically significant change in protein expression between fresh and frozen samples from two independent experiments (Additional file 1: Figure S1A). There was an approximately 25% reduction in the number of viable cells obtained from frozen samples compared to fresh samples (p = 0.048; Additional file 1: Figure S1B). We concluded that using frozen patient samples would require a higher cell count and necessitate the use of a viability dye, but should not affect reliable measurable protein expression determined by flow cytometry.

### Optimization of multiparameter flow cytometry

Use of a multiparameter flow cytometry procedure would minimize introduction of experimental bias and allow endpoint analysis with a fewer net number of patient PBMCs. We optimized a multiparameter flow cytometry procedure and applied it to examine the pattern of expression of γH2AX with MRE11, or with RAD51 in response to O/C treatment. Induction of MRE11 expression was delayed and significantly increased compared to stimulated, but untreated controls after 24 h of treatment, and compared to γH2AX after 48 h of treatment (all p < 0.002; Figure 1e). γH2AX expression increased progressively over time (all p < 0.02; Figure 1e). Both RAD51 and γH2AX showed a significant increase in expression compared to stimulated, but untreated controls after 12 h (all p < 0.002), and RAD51 was significantly higher than γH2AX after 24 h (all p < 0.002). Multiparameter flow cytometry allowed simultaneous evaluation of measures of DNA injury and repair.

### Demonstration of induction of γH2AX and MRE11 by IF

**Figure 2:**
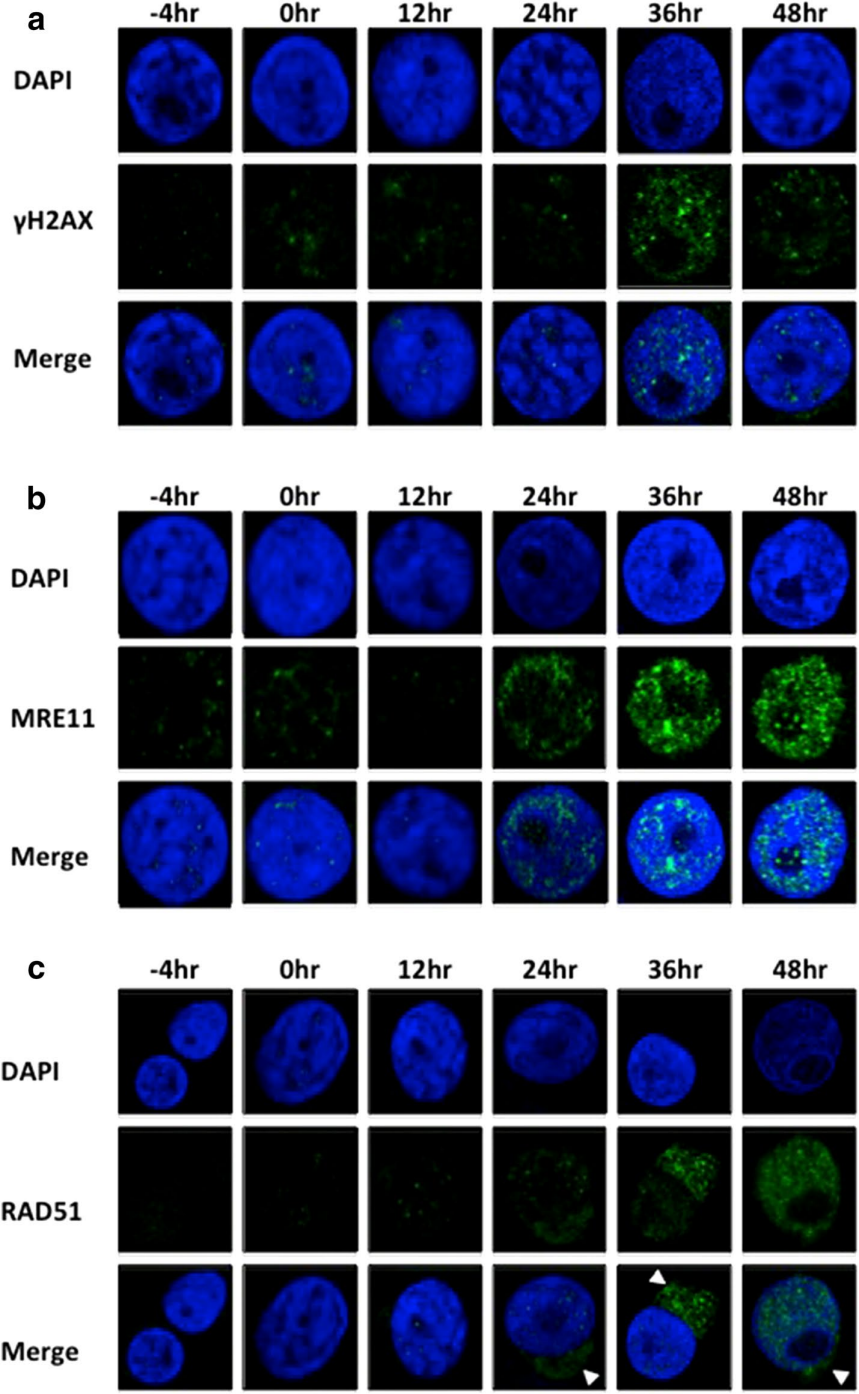
Demonstration of target proteins by IF confocal microscopy. Cells were collected prior to stimulation with PMA/I (−4 h), after stimulation but prior to treatment with O/C (0 h) and every 12 h afterwards for 48 h. **a** γH2AX; **b** MRE11; **c** RAD51.

Immunofluorescence was used to both confirm the temporal and qualitative induction of the signals for γH2AX, RAD51, and MRE11, and also to evaluate the subcellular localization of the IF signal for evaluation of off-target non-nuclear staining that could bias the flow cytometric interpretation. Figure 2 shows representative panels of single stain IF confocal images obtained over the same treatment time course as replicate multiparameter flow cytometric studies. γH2AX and MRE11 (Figure 2a, b) stained nuclear foci, and focus formation increased progressively over injury time. RAD51 foci were both nuclear and cytoplasmic (Figure 2c, arrowheads). Few nuclear RAD51 foci were visible at 12 and 24 h, with discreet perinuclear cytoplasmic foci seen at 24 h, peaking at 36 h. By 48 h, all RAD51 was nuclear. The flow cytometric protocol used cannot distinguish between cytoplasmic and nuclear RAD51 and interpretation of RAD51 results would erroneously include cytoplasmic RAD51 staining as activation in response to injury in a clinical setting where there may not be control for exposure time that this experiment suggests to be necessary. Thus, MRE11 was selected as the marker of DSB repair for analysis of patient PBMCs by multiparameter flow cytometry.

### De novo production of MRE11 occurs in response to treatment with olaparib/carboplatin

**Figure 3:**
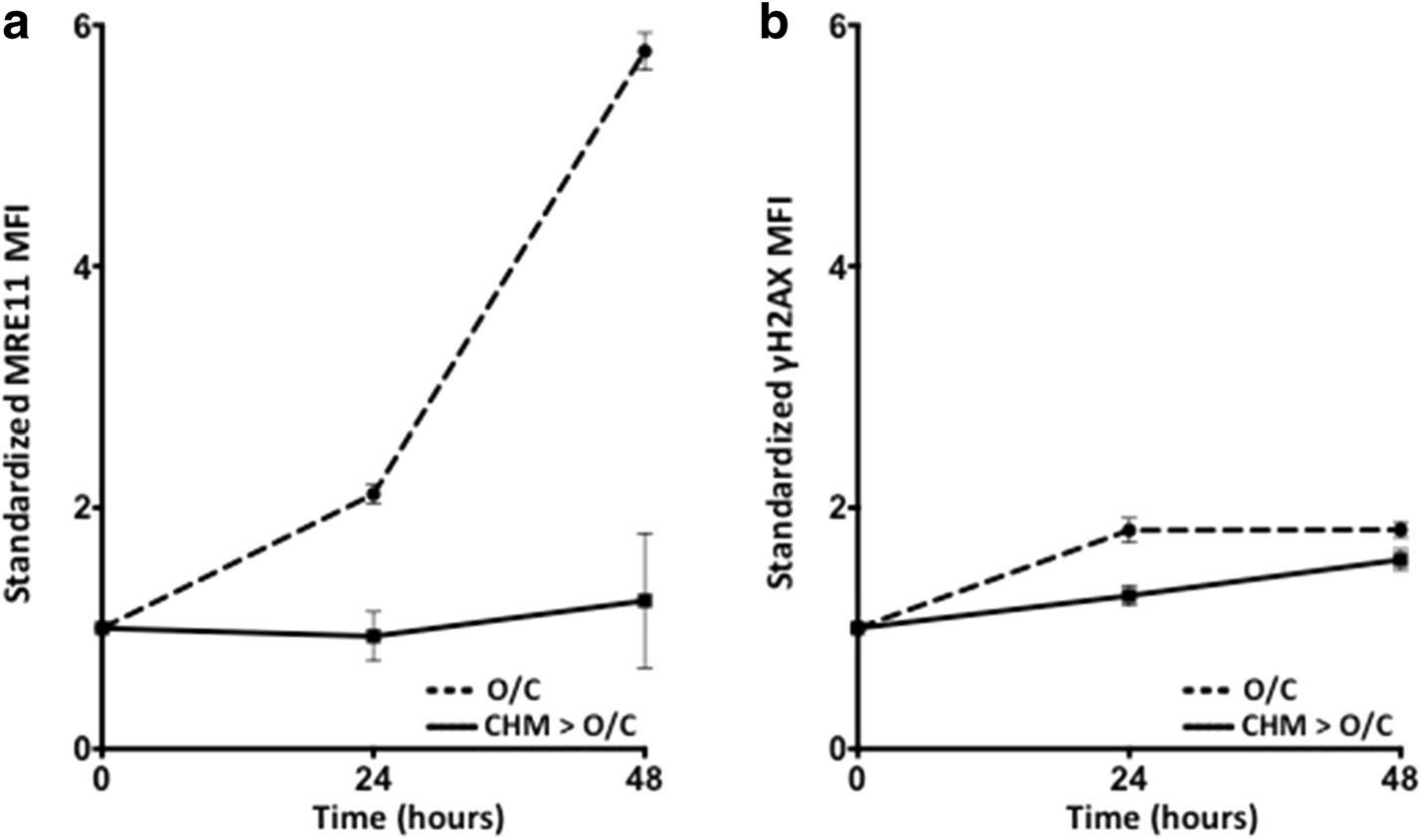
De novo production of MRE11 in response to DNA damage. **a** Cycloheximide (CHM) abolishes MRE11 response to O/C injury. **b** CHM does not inhibit the expression of γH2AX. 24 and 48 h time points compared to 0 time point, and comparison of O/C treatment only (O/C) to CHM followed by O/C (CHM > O/C) for MRE11 or γH2AX at the same time point.

MRE11 is the repair protein recruited in response to DNA injury. The delay in expression of MRE11 detected by flow cytometry seen in Figure 1e raised the possibility of the need for de novo production of MRE11 upon DNA DSB. Cycloheximide (100 μM) was used to block de novo protein synthesis. This concentration inhibits lymphocyte protein synthesis up to 92% with a minimal induction of apoptosis [23]. Figure 3a shows no induction of MRE11 in cells pre-treated with PMA/I in the presence of cycloheximide for 4 h prior to treatment with O/C. Cycloheximide did not have a significant effect on the expression of γH2AX over the same time interval, consistent with the fact that γH2AX arises as the result of post-translational modification and should therefore be unaffected by the inhibition of protein synthesis (Figure 3b). These results indicate that induction of MRE11 protein is a result of exposure to DNA damaging agents and is measurable in stimulated PBMCs.

### Patients who do not respond to PARPi-based therapy have significantly higher baseline levels of γH2AX and MRE11, and a higher ratio of γH2AX/MRE11 compared with responders

**Figure 4:**
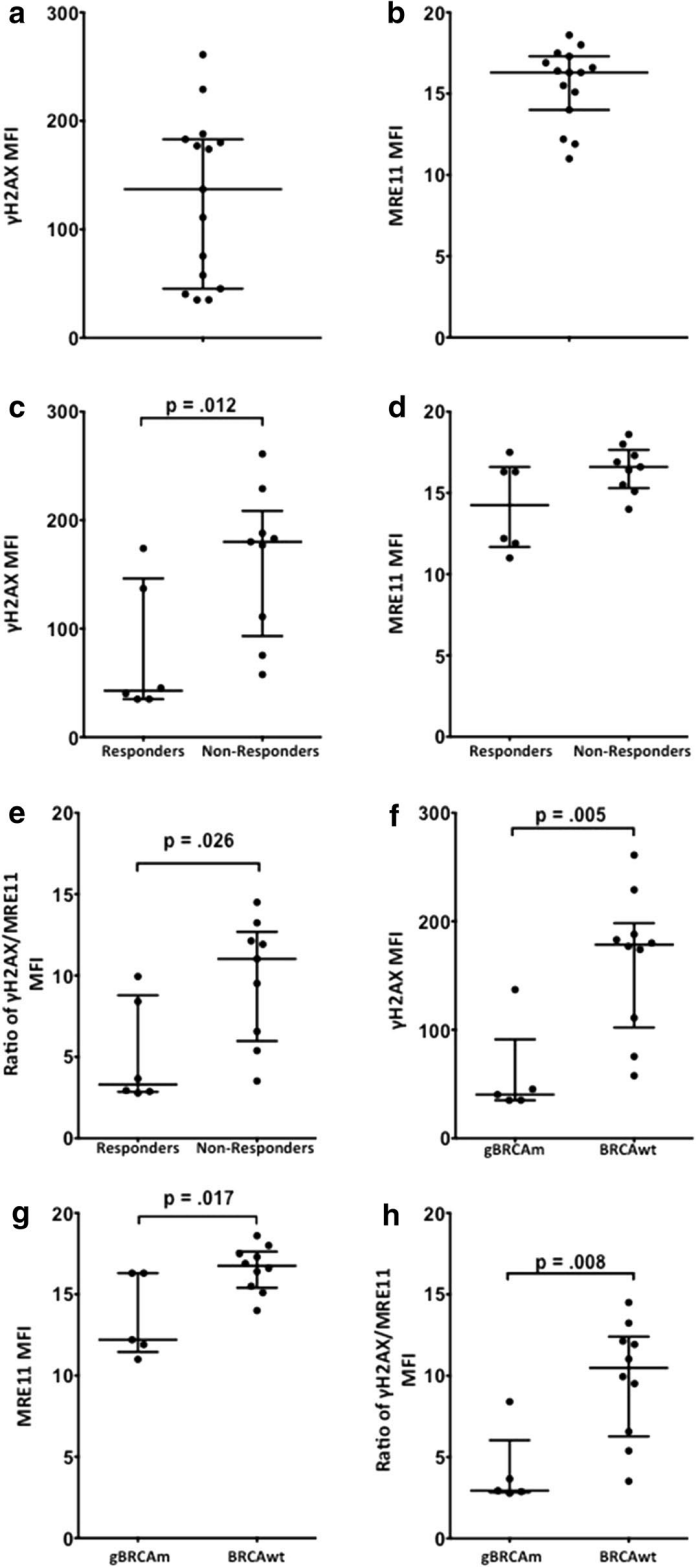
Injury and repair surrogates trend with outcome in PBMCs from women treated with O/C. PBMCs obtained from 15 women pretreated with O/C and viably frozen were available and analyzed according to our demonstrated SOP. The *graphed values* in y axis are raw MFI levels as they represent data from patient samples taken at only one time point (before O/C treatment). **a** γH2AX; **b** MRE11. Median MFI levels of γH2AX (**c**) and MRE11 (**d**) were lower in patients with objective measurable responses to O/C. A pretreatment ratio of γH2AX/MRE11 was significantly higher in patients with no measurable response to O/C (**e**). Pre-treatment γH2AX (**f**), MRE11 (**g**) and a ratio of γH2AX/MRE11 (**h**) by dual-label flow cytometry are lower in gBRCAm patients compared with BRCAwt patients.

We applied our multiparameter flow cytometric γH2AX/MRE11 assay to archival PBMC samples from 15 patients with recurrent HGSOC who received the PARPi, olaparib and carboplatin [22]. Tables 1 and 2 summarize the characteristics and clinical responses of this subset of patients. The global distribution of γH2AX and MRE11 expression across all the patients is shown in Figure 4a, b. In this small group, patients whose best response was stable disease or progression expressed higher pre-treatment levels of both γH2AX and MRE11 compared to patients who had complete or partial responses (p = 0.01 and p = 0.11; Figure 4c, d). We proposed that the ratio of the values of injury to repair, γH2AX/MRE11, may provide a better view of the DNA repair status in the patient PBMCs. The median value of the γH2AX/MRE11 ratio of non-responders was significantly higher compared to responders, indicating more injury than repair (11.0 [range 3.5–13.2] v. 3.3 [range 2.8–9.9], p = 0.026; Figure 4e). Figures 4f–h show the distribution of γH2AX and MRE11 expression and ratio of γH2AX/MRE11 in patients as a function of gBRCAm status. Five of the 15 patients were gBRCAm carriers, and showed significantly lower pre-treatment levels of both γH2AX and MRE11 compared to non-mutation carriers (all p < 0.017; Figure 4f, g). The median value of the γH2AX/MRE11 ratio of BRCAwt was significantly higher compared to gBRCAm carriers (10.5 [range 3.5–13.2] v. 2.9 [range 2.8–8.4], p < 0.01; Figure 4h).

**Table 1:** Patient characteristics

	HGSOC without gBRCAm (10 patients)	HGSOC with gBRCAm (5 patients)
Age in years, median (range)	61 (41–73)	49 (35–57)
Platinum sensitive/platinum-resistant recurrent ovarian cancer, number of patients	3/7	4/1
Number of prior regimens, median (range)	7 (4–12)	5 (4–8)

**Table 2:** Clinical response

Best response	HGSOC with gBRCAm	HGSOC without gBRCAm	Total (N)
	*Median duration of response (range)*	*Median duration of response (range)*	
CR	0	0	0
PR	5/5 (100%) *16* *months (10*–*17* *months)*	1/10 (10%) *3* *months*	6/15 (40%)
SD ≥ 4 months	0/5	3/10 *4* *months (4*–*5* *months)*	3/15 (20%)
PD	0/5	6/10 (60%)	6/15 (40%)

## Discussion

The development of rapidly testable and analytically reliable biomarkers to predict susceptibility to agents targeting HR deficiency is necessary to guide optimal application of PARPi. We hypothesized that quantification of DNA damage and repair using γH2AX and MRE11 or RAD51 may more accurately measure HR potential. We demonstrate the successful development of a multiparameter flow cytometry assay to measure HR status and applied it to a test set of PBMCs from recurrent HGSOC patients receiving a PARPi treatment regimen in a pilot evaluation. Our findings show that patients who do not respond to PARPi-based therapy have significantly higher pretreatment levels of γH2AX and MRE11 and the higher ratio of γH2AX/MRE11, which was opposite of what we expected. We anticipated that non-responders would have the lower ratio of γH2AX/MRE11 due to increased repair function. It is possible that the presence of both underlying DNA damage and increased repair activity are likely due to their prior exposure to DNA damaging agents and other cytotoxic chemotherapies prior to study enrollment and our results suggest greater baseline injury than repair may reflect a potential mechanism of resistance.

Approximately 50% of HGSOC tumors are proposed to have some deficiency in HR, due to gBRCAm or acquired defects in proteins of the HR pathway [24]. RAD51 is a DNA recombinase that interacts with BRCA2 and other HR proteins upon DNA injury [25]. DNA DSB are resected to generate single stranded (ss) DNA tails and the RAD51-ssDNA filaments continue to grow from a BRCA2-stablized filament nucleus to form an ATP-bound nucleoprotein filament, capable of homologous DNA pairing [26]. Quantification of RAD51 nuclear focus formation has been used to assess HR competence in epithelial ovarian cancer ascites primary cultures, and was correlated with response to PARPi in vitro [12]. Dedes et al. demonstrated a correlation between reduced RAD51 nuclear focus formation and PARPi sensitivity in PTEN-deficient endometrial cancer cell lines in in vitro [27]. Low quantities of RAD51 foci measured by IF in post-chemotherapy biopsies were associated with pathologic complete response to anthracycline-based chemotherapy in sporadic primary breast cancers [28]. However, none of these studies examined if the presence of RAD51 foci prior to or early in therapy could predict clinical benefit. None used a method that could be applied readily in a more high throughput fashion. While our findings showed RAD51 could be measured by flow cytometry and reflected injury, our confirmation of concomitant cytosolic expression during the time frame of injury indicates that a flow cytometry method would erroneously count cytoplasmic RAD51 staining as positive for repair in response to injury. Therefore, any measure of RAD51 as a predictive biomarker would need to control for subcellular localization as a function of time.

MRE11 is a nuclease that facilitates the generation of 3′ overhangs by making a DNA nick at a point separate from the break ends, creating an entry site for further processing by exonuclease enzymes [29–31]. The 5′-terminated DNA strand must first be resected to generate a 3′ single-stranded DNA overhang for repair by HR [31]. The MRE11/RAD50/NBS1 (MRN) complex binds tightly to chromatin in the absence of DNA damage during S phase [32]. HR is activated and works during the S and G2 phases of the cell cycle [33]. Martin et al. showed MRE11 is induced in response to DNA injury and co-localized with γH2AX after irradiation, measured by IF in lymphoblastoid cell lines in vitro [34]. Choudhry and colleagues examined protein expression of MRE11, RAD50, NBS1, ATM, and H2AX by immunohistochemistry in pretreatment tumor specimens from 179 bladder cancer patients receiving radical radiotherapy. They showed low MRE11 protein expression was associated with worse cancer-specific survival following radiotherapy [14]. We confirm that MRE11 is a preferable measure of DNA DSB repair in a flow cytometry high throughput approach, as it is selectively nuclear. Our finding of cycloheximide-sensitive induction of MRE11 indicates that in this model system, new MRE11 protein is induced secondary to cellular recognition of DNA injury. This further suggests that MRE11 may be a more useful marker, as it is responsive to dynamic cellular events. It has been reported that MRE11 recruitment at the sites of DNA damage is rapid and detectable by IF within 10–20 min after irradiation in human fibroblasts [35–37]. A small subset of HR deficient cancers have been found to have MRE11 mutations providing a rare confounder while supporting the potential of low MRE11 as a biomarker of HR behavior [38, 39].

Flow cytometry offers the possibility to quantify γH2AX or MRE11 expression in large cell populations [40, 41]. The flow cytometric approach to measure DNA damage is rapid, more sensitive, and less cumbersome compared with alternative, commonly used methods, including the comet assay for single cell DNA damage assessment or IF analysis of γH2AX or MRE11 nuclear foci [42, 43]. The use of dual staining may have resulted in a reduction in dynamic range in the flow cytometry assay. This may be due to use of different antibodies and detection reagents. The flow cytometric assay reported here requires only 2 mL of whole blood and can be performed with as few as 10^5^ PBMCs [42]. In a study by Wilkins et al., flow cytometric analysis of the level of γH2AX in PBMCs was examined in prostate cancer patients as a potential marker of radiosensitivity, which was defined by radiation therapy-induced proctitis. That study which looked at γH2AX alone found no correlations of γH2AX expression by flow cytometry and radiation sensitivity [44]. Our pilot study shows that both DNA injury and repair can be measured and correlated in single cells by high-throughput flow cytometry.

Preanalytical variables are important in biomarker reliability. It is possible that there may be false positive γH2AX or MRE11 staining due to cell injury caused during PBMC freezing and storage. Our findings indicate that the freezing and thawing process affected cell viability, but did not result in a significant differences in expression of γH2AX, MRE11 or RAD51 compared to paired fresh samples. In addition, the flow cytometry assay includes two approaches to gating out dead cells to overcome this issue. Light scatter properties and use of the LIVE/DEAD Fixable Aqua dead cell stain allow confidence in target measurements in live cells.

Our study has some limitations. First, a small patient sample size may introduce biases in interpreting the relationship between the flow cytometry results and clinical responses. Second, this post hoc study of 15 patients was an exploratory objective, and the study sample size was not powered to address this question, thus it is possible that a dual-label flow cytometry assay may not predict the clinical response. Third, it should be stressed that despite our findings related to the sensitivity to PARPi based therapy, the majority of responders were gBRCAm carriers and all patients were heavily pretreated with cytotoxic chemotherapies prior to study enrollment. Nevertheless, it is important to note that not all gBRCAm-associated HGSOC patients respond to PARPi-based therapy and our assay could be complementary to guide the PARPi treatment in HR-deficient HGSOC with gBRCAm carriers. The exploratory nature of our study requires that these findings be examined in a prospective and powered setting before definitive conclusions can be drawn.

Collection of PBMCs as a surrogate tissue to monitor DNA damage response has several advantages over tumor biopsy or circulating tumor cells (CTCs), including minimally invasive sample collection, the ability to collect multiple samples for longitudinal assessment of drug effect, and a reliable yield of a number of cells since isolation and enumeration of CTCs are often challenges in HGSOC [45, 46]. It has been proposed that the in vitro radiosensitivity of PBMCs correlates with individual sensitivity to ionizing radiation in breast cancer patients, and the in vitro radiosensitivity of PBMCs is higher in lung, head and neck, and breast cancer patients compared to healthy donors [18, 47–49]. It is possible that a different composition of lymphocyte subpopulation of PBMC was collected from cancer patients compared to healthy donors, and this difference may not reflect tumor per se. Quantification of γH2AX and MRE11 at the single-cell level by multiparametric flow cytometry using CTCs or tissue samples may be considered as another application.

There are now a number of PARPi in phase I to III clinical investigation in gBRCAm associated- ovarian cancer and other solid tumors. There is a need to develop a functional assay to reflect HR potential that is feasible and applicable in clinical settings to facilitate individualizing therapy. HR deficiency may identify a distinct patient population with favorable biologic features for HR-targeted therapy not only in the context of HGSOC but also in other cancer types. Our data present an opportunity to apply a simple, high-throughput method using easily obtainable PBMCs, and our exploratory findings, though provocative, require sufficiently powered prospective studies.

## Conclusions

Our results demonstrate the successful development and application of multiparameter flow cytometry measuring γH2AX and MRE11 in PBMCs and support further of this assay as a potential predictive biomarker to PARPi-based therapy.

### Additional file

Additional file 1: Figure S1. Protein expression and cell viability in frozen cells. **(A)** Freezing and thawing cells have no statistically significant effects on protein expression for γH2AX, MRE11 or RAD51 (all p ≥ 0.4). **(B)** Cell viability is significantly reduced as a result of the freezing/thawing process.

## Abbreviations

PARPi: poly(ADP-ribose) polymerase inhibitors
HR: homologous recombination
HGSOC: high-grade serous ovarian cancer
DSB: DNA double-strand breaks
gBRCAm: deleterious germline *BRCA1* and *BRCA2* mutation
BRCAwt: *BRCA* wild-type
RR: response rate
PBMCs: peripheral blood mononuclear cells
IRB: Institutional Review Board
PMA: phorbol myristate acetate
I: ionomycin
DMSO: dimethyl sulfoxide
FBS: fetal bovine serum
PBS: phosphate buffered saline
RPMI-1640 medium: Rosewell Park Memorial Institute-1640 medium
MFI: median flow intensity
SEM: standard error of the mean
CTCs: circulating tumor cells
CHM: cycloheximide
SOP: standard operating procedure
FITC: fluorescein isothiocyanate
APC-A: allophycocyanin-A
