# Supplementary figures and images for: Development of a multiparameter flow cytometric assay as a potential biomarker for homologous recombination deficiency in women with high-grade serous ovarian cancer
Source: J Transl Med. 2015 Jul 22;13:239. doi: 10.1186/s12967-015-0604-z (PMC4508767; doi:10.1186/s12967-015-0604-z)

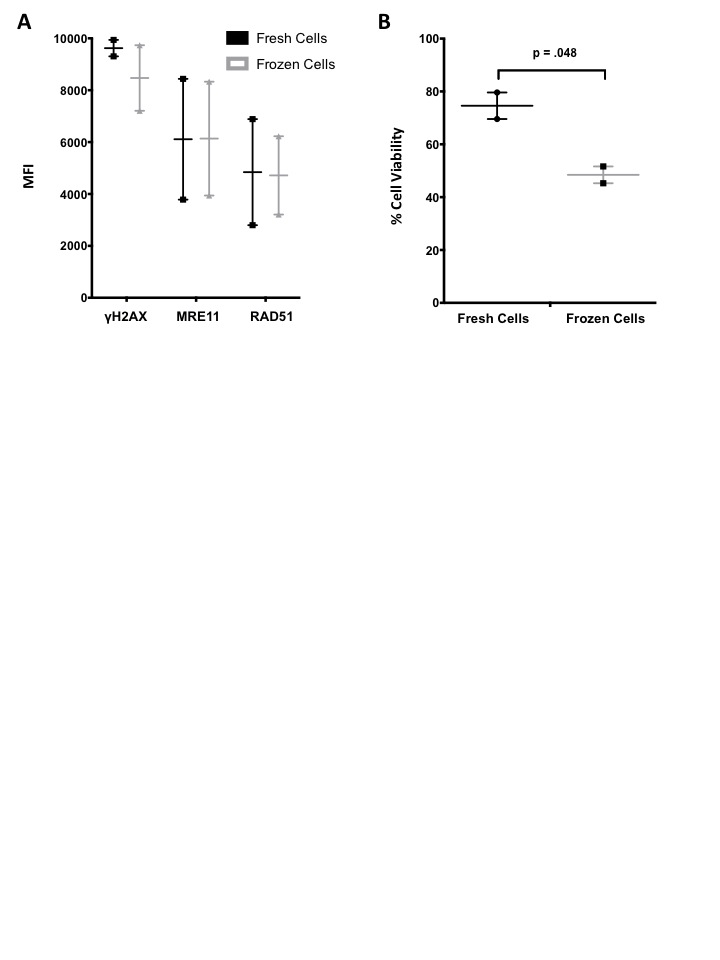

Supplement: Supplementary file 1 — Protein expression and cell viability in frozen cells. (A) Freezing and thawing cells have no statistically significant effects on protein expression for γH2AX, MRE11 or RAD51 (all p ≥ 0.4). (B) Cell viability is significantly reduced as a result of the freezing/thawing process. [file 12967_2015_604_MOESM1_ESM.jpg]
